# Single dose triple drug therapy completely suppresses *Wuchereria bancrofti* microfilaremia for 5 years

**DOI:** 10.1056/NEJMc1914262

**Published:** 2020-05-14

**Authors:** Christopher L. King, Gary J. Weil, James W. Kazura

**Affiliations:** 1 Center for Global Health and Diseases, Case Western Reserve University School of Medicine and Veterans Affair Medical Center, Cleveland, OH; 2 Department of Medicine, Infectious Diseases Division, Washington University School of Medicine, St. Louis, MO; 3 Center for Global Health and Diseases, Case Western Reserve University School of Medicine, Cleveland, OH

Lymphatic filariasis is a neglected tropical disease caused by the nematode parasites *Wuchereria bancrofti* and *Brugia spp*. that is targeted for elimination by mass drug administration (MDA)^1^. In 2018, we published results from a clinical trial showing a single co-administered dose of ivermectin/diethylcarbamazine/albendazole (IDA) cleared microfilaremia in 55 of 57 individuals with bancroftian filariasis for three years after treatment^2^. This result was far superior to that obtained with the prior standard two-drug diethylcarbamazine/albendazole regimen. Consequently WHO modified their guidelines to recommend IDA therapy for filariasis elimination outside of sub-Saharan Africa in regions that have not started or delivered fewer than four annual rounds of diethylcarbamazine/albendazole or have not met thresholds for transmission interruption^3^. Merck Inc. expanded its ivermectin donation by 100 million additional doses annually to help facilitate this change, with 68 million people expected to receive IDA next year.

One limitation of IDA treatment was that most individuals did not completely clear circulating filarial parasite antigen^2^(a biomarker for living adult filarial worms^4^), suggesting that IDA sterilized adult worms without killing all of them. Since the estimated reproductive lifespan of filarial adult worms is five years^5^, it was possible that remaining worms might recover and start producing microfilaria. To investigate whether IDA had sterilized adult worms, we reexamined 36 individuals approximately 5 years after single dose IDA treatment (two years after the completion of the clinical trial) using the same parasitological methods described in the original study^2^. None of these individuals had received any subsequent treatment for lymphatic filariasis. 35 of 36 individuals had zero microfilaria in 2 ml of venous night blood (Figure 1). One person had a single microfilaria in 2 mL of blood, which is far below the concentration required for sustaining transmission by local mosquitos. However, only 9 of 36 participants (25%) had negative filarial antigen tests at five years. There was no difference in age, sex and baseline microfilaremia levels in the 36 individuals retested at five years and 19 that were not retested. MDA and distribution of insecticide-treatment bednets in the study area may explain the lack of reinfection in study participants in the five years after IDA treatment. These data support the hypothesis that IDA sterilizes adult filarial worms for at least five years but often fails to clear circulating filarial antigen. Thus, a better biomarker or a different surveillance strategy will be needed for assessing the impact of IDA on lymphatic filariasis populations.

**Figure legend:**
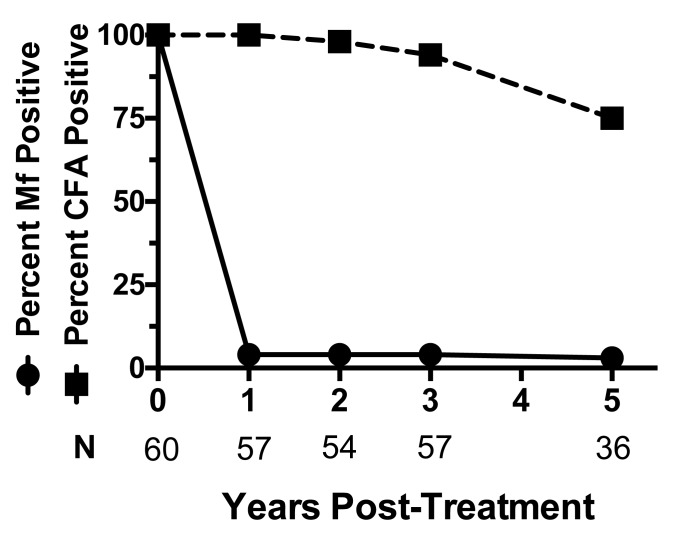
Single dose IDA treatment totally cleared microfilaremia in 97% of individuals (dark circles) for 5 years. The baseline geometric mean blood microfilaria (Mf) count was 699 microfilaria/mL (range 55-15,621). Triangles show the percentages of individuals that remained circulating filarial antigen (CFA) positive.
